# Full Coverage Testing Method for Automated Driving System in Logical Scenario Parameters Space

**DOI:** 10.3390/s25185764

**Published:** 2025-09-16

**Authors:** Haitao Min, Zhiqiang Zhang, Tianxin Fan, Peixing Zhang, Cheng Zhang, Ge Qu

**Affiliations:** 1 National Key Laboratory of Automotive Chassis Integration and Bionics, Jilin University, Changchun 130025, China; minht@jlu.edu.cn (H.M.);; 2 China Automotive Technology & Research Center (Tianjin) Co., Ltd., Tianjin 300399, China

**Keywords:** automated driving system, test scenario, full coverage testing, concrete scenario representativeness

## Abstract

Scenario-based testing is a mainstream approach for evaluating the safety of automated driving systems (ADS). However, logical scenarios are defined through parameter spaces, and performance differences among systems under test make it difficult to ensure fairness and coverage using the same concrete parameters. Accordingly, an automated driving system testing method is proposed. Guided by the established full-coverage testing framework, a quantitative evaluation method for scenario representativeness is first proposed by jointly analyzing naturalistic driving probability distributions and hazard-related characteristics. Furthermore, a hybrid algorithm integrating heat-guided hierarchical search and genetic optimization is developed to address the non-uniform full-coverage problem, enabling efficient selection of representative parameters that ensure complete coverage of the logical scenario space. The proposed method is validated through empirical studies in representative use cases, including lead vehicle braking and cut-in scenarios. Experimental results show that the proposed method achieves 100% coverage of the logical scenario parameter space with an 8% boundary fitting error, outperforming mainstream baselines including monte carlo (84.3%, 19%), combinatorial testing (86.5%, 14%) and importance sampling (72.0%, 7%). The approach achieves exhaustive coverage of the logical scenario space with limited concrete scenarios, and effectively supports the development of consistent, reproducible and efficient scenario generation frameworks for testing organizations.

## 1. Introduction

Scenario-based testing has gradually become the mainstream approach for evaluating the performance of automated driving systems (ADS) [1,2,3]. In this framework, logical scenarios are described using parameter spaces, forming the foundation of scenario library construction and serving as key references for third-party testing organizations in defining test boundaries [4,5]. However, during the performance validation of different systems, testing organizations are required to establish standardized and regulated concrete scenario libraries to enable comprehensive evaluation of the logical scenario parameter space [6]. The generation of such concrete scenarios must ensure both consistency and coverage, which has become a major obstacle for third-party testing practices.

Existing concrete scenario generation methods can be broadly categorized into two types: online optimization methods aimed at identifying the performance boundaries of the system under test, and offline optimization methods intended to generate test case libraries [7].

Online optimization methods embed the system under test into a closed-loop simulation environment and accelerate the identification of performance boundaries through techniques such as optimization-based search and importance sampling. Representative examples include the method proposed by Feng for generating hazardous scenarios based on improved optimization algorithms [8], the logical scenario boundary search approach developed by Mullins et al. [9], the accelerated crash rate evaluation method based on importance sampling introduced by the University of Michigan [10], and Yang et al. proposed an adaptive testing framework that integrates deep learning with importance sampling, aiming to continuously optimize the testing strategy and evaluate the safety performance boundaries [11]. These methods generally rely on optimization algorithms as the core, generating scenario parameters in real time during testing via probabilistic sampling. Due to the randomness inherent in the sampling process and the performance differences across systems, the sampled concrete parameters often differ significantly between systems [12,13]. As a result, such methods face challenges in meeting the consistency requirements of third-party testing and in ensuring full coverage of the logical scenario parameter space [14].

Offline optimization methods do not take into account the performance differences among systems under test. Instead, they rely on uniform or random sampling to generate identical sets of concrete scenarios for constructing scenario libraries and carrying out evaluations, thereby aligning with the consistency requirements of third-party testing organizations. Li et al. presents an open-source tool that leverages constraint-aware k-way combinatorial testing and scenario instantiation to efficiently generate diverse, high-coverage test scenarios [15]; Mancini enabled the random sampling or enumeration of input scenarios of arbitrary length under complex constraint conditions, thereby enhancing the efficiency and practicality of the system verification process [16]; Li et al. proposed a weight-guided combinatorial testing method for generating simulated autonomous driving scenarios based on operational design domain elements [17]; Duan et al. introduced a parameter generation method based on combinatorial complexity [18]; Gambi et al. developed a monte carlo-based strategy for test case creation [19]; and Li et al. presented a scenario generation technique using Latin Hypercube Sampling [20]. While these methods offer consistency in logical scenario sampling, they typically depend on predefined parameter discretization steps. A large step size may hinder complete space coverage, while an overly small one can lead to excessive test cases and reduced testing efficiency [21,22].

To address the limitations of existing methods, this paper proposes a full-coverage testing approach for the logical scenario parameter space of automated driving systems, centered on scenario representativeness. A quantitative evaluation model for assessing the representativeness of concrete scenarios is established, and a corresponding non-uniform space coverage strategy is developed. By selecting a limited number of representative concrete scenarios, complete coverage of the logical scenario space is achieved, while simultaneously ensuring reproducibility and consistency of test results, along with high coverage and efficient execution.

A full-coverage testing framework for the logical scenario parameter space of automated driving systems is first proposed, formulated based on the principle of scenario representativeness. A quantitative evaluation method is developed to assess scenario representativeness by jointly considering hazardousness and the probability distribution derived from naturalistic driving data. To address the optimization problem of achieving full coverage in a differentiated sample combination space, a greedy algorithm-based approach is introduced to identify the minimal set of concrete scenarios capable of covering the entire parameter space. The proposed method is validated using two typical cases: lead vehicle braking and lead vehicle cut-in scenarios. Experimental results demonstrate that the proposed approach achieves 100% coverage of the logical scenario parameter space and maintains a boundary fitting error of 8%. In comparison, the monte carlo method achieves 84.3% coverage with a fitting error of 19%, while the combinatorial testing method reaches 86.5% coverage and a fitting error of 14%.

## 2. Full-Coverage Testing Framework

**Figure 1:**
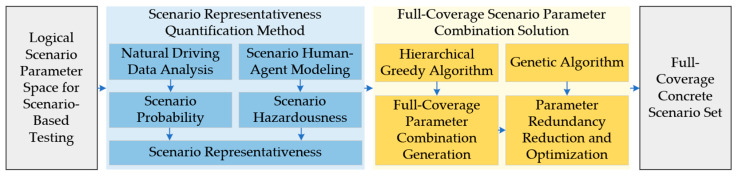
Framework for Full-Coverage Testing of Automated Driving Systems.

Full-coverage testing of the automated driving system under test within the logical scenario parameter space is achieved through a concrete scenario generation method, as illustrated in Figure 1.

Assuming uniform system performance across the logical scenario parameter space, the representativeness of a concrete scenario should be determined by two inherent attributes: probability and hazardousness. A quantitative evaluation method for scenario representativeness is initially established, incorporating both scenario probability and scenario hazardousness, and then operationalize representativeness as an influence range that quantifies the size of the scenario’s substitutable neighborhood in the parameter space. The scenario probability is derived from the statistical distributions of key parameters observed in naturalistic driving data [23], while the scenario hazardousness is estimated through the construction of a driver surrogate model. Based on the computed representativeness, a heat-guided hierarchical greedy algorithm is employed to extract a set of concrete scenarios that fully cover the parameter space. To further reduce redundancy, a genetic algorithm is applied to eliminate overlapping scenario instances, resulting in a more compact yet complete concrete scenario test set.

## 3. Quantitative Method for Scenario Representativeness

Within the logical scenario parameter space, assuming no performance heterogeneity of the system under test, the representativeness of a concrete scenario is jointly determined by two intrinsic attributes: probability and hazardousness. The former refers to the frequency with which a given scenario occurs in real-world naturalistic driving, while the latter reflects the extent to which the scenario is associated with safety-critical events or collisions. Scenarios that are both high-risk and frequently observed in naturalistic datasets play a crucial role in safety validation. Due to their critical nature, such scenarios cannot be easily substituted by neighboring cases, and therefore should be assigned higher testing priority and density. In coverage calculation, they should be associated with smaller influence radii and thus lower representativeness values. In contrast, scenarios that are low-risk or rarely encountered may be covered using broader influence regions to reduce redundant sampling. Accordingly, these scenarios are considered to have higher representativeness.

### 3.1. Scenario Representativeness Model

Building upon the above conceptual foundation of scenario representativeness, a risk–probability jointly driven representativeness quantification function is proposed. This function adaptively determines the influence range of each concrete scenario within the parameter space, thereby providing a structurally sound spatial constraint for subsequent test point selection and coverage optimization.

The quantitative evaluation of concrete scenario representativeness proposed in this study is defined in Equation (1).

(1) $$r_{i}^{\left(j\right)}{=\omega }_{j}^{-1}\times \varphi \times {\left(\frac{\max\zeta }{\zeta_{i}}\right)}^{\sigma }\times {\left[log\left(\frac{1}{E_{i}+\tau }+1\right)\right]}^{\lambda }$$

In this equation, *r_i_*^(*j*)^ denotes the influence radius of the *i* concrete scenario along the *j* dimension of the parameter space, which reflects its representativeness. *ζ_i_* represents the scenario hazardousness index of the *i* scenario (shown in Section 3.3), and *E_i_* denotes its scenario probability index (shown in Section 3.2). *σ* denotes the hazardousness scaling exponent, which governs the weight of hazardousness in the representativeness computation. *λ* denotes the scaling exponent applied to the probability function, governing the extent to which scenario occurrence probability modulates the representativeness radius. *τ* is a probability regularization constant, used solely to prevent logarithmic divergence when *E_i_* approaches zero. *φ* denotes the global influence-range scaling coefficient, which linearly rescales all computed radius to align with the size of the specific logical parameter space, the discretization step, and external constraints such as testing computational budget. *ω_j_* is the weight coefficient for the *j* dimension.

In Equation (1), since the logical scenario parameter space is typically discretized with fixed step sizes, the distances between adjacent test points are uniformly partitioned. If continuous or fractional influence radii are used, it may result in ambiguous boundaries regarding whether neighboring points are covered, thereby reducing the model’s operational feasibility and stability. To ensure controllable structural boundaries and computational cost during test point selection and space search, the influence radius *r_i_*^(*j*)^ is constrained to take integer values by rounding down. Additionally, a global minimum value *r*_min_ is introduced to avoid degenerate point-wise influence regions caused by overly high probability or extremely low risk. A maximum value *r*_max_ is also defined to prevent excessive expansion of the influence range in cases where corner scenarios have extremely low probabilities. The normalization term (*ζ_i_*\max*ζ*)*^σ^* is introduced to scale the hazardousness level of each scenario, enabling more pronounced contraction of the influence radius for high-risk cases while mitigating inconsistencies in influence range across different logical scenarios caused by variations in risk level configurations. For the parameter *ω_j_*, the impact of each scenario parameter dimension on testing outcomes varies significantly. This inter-dimensional heterogeneity manifests as distinct risk gradients within the parameter space. Accordingly, independent weight coefficients *ω* are introduced into the adaptive influence radius function to modulate expansion capability along each dimension, allowing the influence range to more precisely reflect the actual relevance of each scenario parameter. It is important to note that the actual logical scenario parameter space comprises both continuous and discrete parameters. The former exhibit numerical continuity and are thus suitable for coverage-based representativeness analysis. In contrast, discrete parameters are defined through enumeration and lack direct numerical comparability across dimensions. To address this, discrete parameter combinations are treated as categorical labels to partition the entire logical scenario parameter space into multiple sub-scenarios. Representativeness evaluation and scenario selection are then independently performed within each sub-scenario, followed by result integration. This hierarchical strategy avoids structural interference in the model caused by cross-type variable mixing.

### 3.2. Scenario Probability Index

For the scenario probability *E* in Equation (1), a Gaussian distribution model is adopted in this study. In real-world traffic behavior, driver responses to environmental changes exhibit certain regularity and concentration, where most control and dynamic parameters tend to cluster around typical values. This characteristic supports the assumption that parameter samples collected from naturalistic driving conform statistically to a normal distribution [24]. Accordingly, the Gaussian model is employed to characterize the probability distribution of test scenarios within the logical parameter space. The computation of the scenario probability index *E* is defined in Equation (2).

(2) $$E_{i}=\frac{1}{\sqrt{{\left(2\pi \right)}^{\vartheta }\left|\Sigma \right|}}\exp\left[-\frac{1}{2}{\left(X-\eta \right)}^{T}\Sigma^{-1}\left(X-\eta \right)\right]$$

In this equation, *ϑ* denotes the dimensionality of the parameter space, *Σ* is the covariance matrix characterizing the correlation among different types of parameters, *η* represents the mean vector of all parameter variables, and *X* denotes the concrete scenario parameter vector.

### 3.3. Scenario Hazardousness Index

For the scenario hazardousness index *ζ* defined in Equation (1), both collision and non-collision cases are considered. For collision scenarios, hazardousness is categorized into three levels based on the relative velocity between vehicles at the moment of impact: severe collisions correspond to relative velocities greater than 65 km/h, moderate collisions range from 20 km/h to 65 km/h, and minor collisions are defined as those below 20 km/h [25]. For non-collision scenarios, the hazardousness index is computed based on the inverse of the time to collision (*TTC*), as described in Equation (3).

(3) $${\mathrm{TTC}}^{-1}=\frac{\Delta v}{\Delta dis}$$

In this equation, Δ*dis* denotes the longitudinal distance between the ego and leading vehicle, and Δ*v* represents their relative velocity. Existing studies generally regard *TTC* values below 1.5 s as indicative of an imminent collision risk [26]. In this study, 0.7 s^−1^ is adopted as the *TTC*^−1^ boundary between near-collision and safe scenarios.

By jointly considering both collision and non-collision scenarios, the scenario hazardousness index *ξ* is defined in this study using a five-level risk scale: severe collisions are assigned a value of 1.0, moderate collisions 0.8, minor collisions 0.6, near-collision scenarios 0.4, and safe scenarios 0.2.

Due to the difficulty of directly estimating the scenario hazardousness index from existing data through statistical means, this study introduces a surrogate modeling approach to infer the hazardousness values of previously unobserved scenarios.

The driver surrogate model comprises longitudinal and lateral components. The longitudinal behavior is modeled using a long short-term memory (LSTM) neural network, while the lateral behavior is simulated using a lane-changing potential field model in combination with monte carlo tree search (MCTS).

**Figure 2:**
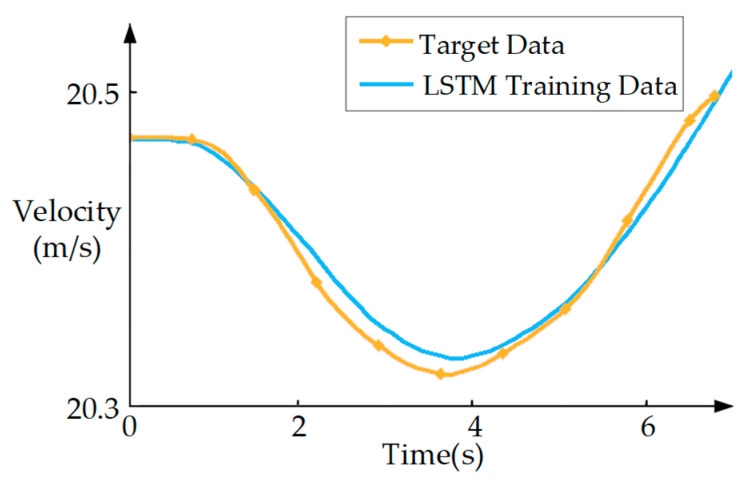
Control Performance of the Longitudinal Surrogate Model.

The longitudinal model is trained using the publicly available HighD dataset, with the training parameters detailed in Table 1. The corresponding training results are illustrated in Figure 2.

To quantify the discrepancy between the longitudinal surrogate model and actual vehicle dynamics, the root mean square error (RMSE) is employed as the evaluation metric for model accuracy. The resulting RMSE is 0.030, which meets the fidelity requirements for surrogate modeling in this study.

For the lateral surrogate model, the lane-changing potential field quantifies the driver’s “satisfaction” with the current traffic environment and provides a dynamic intention input for lane-change planning. MCTS explores optimal lane-changing strategies by simulating multiple possible maneuver trajectories.

The lane-changing potential field model defined in this study incorporates multiple factors, including vehicle speed, mass, relative motion with the leading vehicle, and road adhesion conditions. The computation is formulated in Equation (4). In this framework, the threshold value *B* for generating a lane-change intention is set to 0.15.

(4) $$B=\frac{I\times T\times M_{i}}{\Delta {\mathrm{dis}}_{\mathrm{ij}}^{l_{1}}}\times \exp\left[l_{2}\times \Delta v\times {\cos\theta }_{\mathrm{ij}}\right]$$

In this equation, *I* is a constant used to adjust the magnitude of the potential field, *M_i_* denotes the equivalent mass of the target vehicle, *l*_1_ and *l*_2_ are exponential decay factors used to characterize the influence of distance and velocity on the potential field, these specific values of these parameters are provided in Table 2. Δ*dis_ij_* represents the relative distance between the ego vehicle and the target vehicle, Δ*v* refers to the relative velocity between the ego vehicle’s speed and that of the target, the angle *θ_i_* denotes the angle between the ego vehicle’s velocity vector and the line connecting the ego and target vehicles.

In Equation (4), the environmental perception weight *T* is adjusted based on the road adhesion coefficient under different driving conditions. Under low-adhesion scenarios, drivers tend to actively reduce vehicle dynamic responses, thereby attenuating the accumulation of potential. This adjustment is formulated in Equation (5).

(5) $$T={\left(1\;{+e}^{-\rho \left(\mu -\mu_{0}\right)}\right)}^{-1}$$

In this formulation, *ρ* is a constant used to modulate the influence of the environmental perception weight, and *μ* denotes the road adhesion coefficient.

Upon the generation of a lane-change intention, MCTS is employed to explore the optimal lane-change trajectory [27]. The MCTS process comprises the following four steps:

1. Selection: Starting from the root node, the most promising child node is selected by traversing the monte carlo tree. The selection aims to maximize the estimated value of the decision strategy.
2. Expansion: If the selected node is not fully expanded (i.e., it does not yet contain all possible child nodes), it will be expanded by generating one or more new child nodes.
3. Simulation: From the newly expanded node, a rollout is conducted by simulating future lane-change paths in a stochastic manner to estimate their expected cumulative return.
4. Backpropagation: The simulation result is propagated back from the leaf node to the root node, updating the reward statistics along the traversed path.

Through repeated simulations of various lane-change paths and iterative backpropagation, the MCTS algorithm evaluates each trajectory and progressively refines the node values, ultimately converging on an optimal lane-change plan.

**Figure 3:**
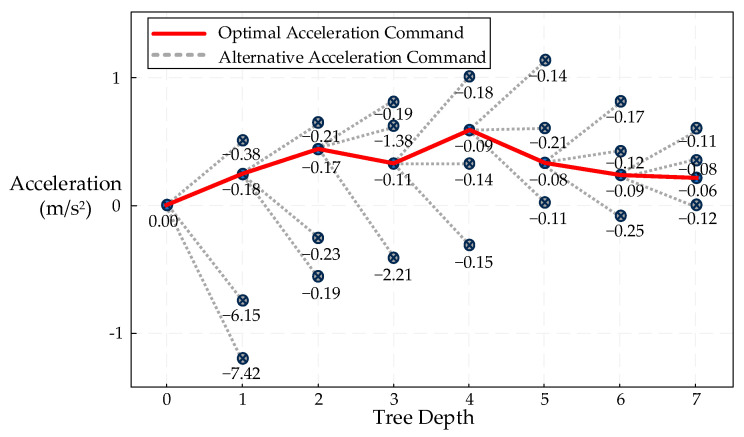
Illustration of the MCTS Performance.

The illustration of the MCTS result is presented in Figure 3.

## 4. Computation of Fully Covering Scenario Parameter Sets

A heat-guided hierarchical greedy coverage optimization (H-GCO) algorithm is proposed to achieve full coverage of the logical scenario parameter space. This algorithm evaluates the marginal coverage gain of candidate scenario parameter combinations and incrementally generates representative sets in a block-wise manner across the space. On this basis, a genetic algorithm-based compression strategy is further introduced to minimize the total number of scenario combinations required for coverage, thereby enhancing the efficiency of automated driving system validation under the constraint of complete parameter space coverage.

### 4.1. Generation of Scenario Parameter Sets for Full Coverage

**Figure 4:**
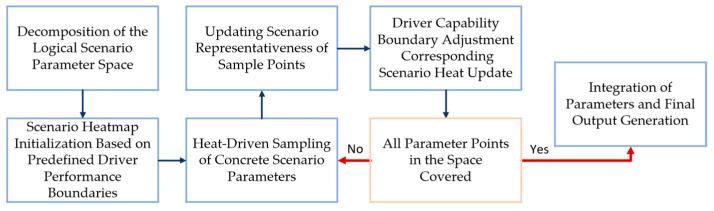
Workflow for Generating Full-Coverage Scenario Parameter Sets.

The greedy algorithm is a heuristic approach that iteratively constructs a near-optimal global solution by making locally optimal choices at each step. In each iteration, the element that yields the highest marginal gain from the current candidate set is added to the solution set. Based on this principle, the heat-guided hierarchical greedy algorithm is proposed to generate a concrete scenario set that achieves full coverage of the logical scenario parameter space. The overall procedure is illustrated in Figure 4.

Step 1: The entire logical scenario parameter space is uniformly partitioned into multiple sub-regions. A greedy search is independently performed within each local region.

Step 2: For each sub-region, scenario heat values are computed for all concrete parameter combinations based on a predefined boundary of driver capability, serving as a prior to estimate the potential criticality of each scenario.

Step 3: For each candidate scenario, the cumulative heat within a fixed-radius neighborhood is calculated. The scenario located at the center of the neighborhood with the highest cumulative heat is selected as the current sampling point.

Step 4: The surrogate model is used to evaluate the actual representativeness of the selected sampling point.

Step 5: Based on the surrogate evaluation, the pre-defined driver capability boundary is updated, and the scenario heat is recalculated accordingly.

Step 6: Steps 3 through 5 are repeated iteratively until the entire parameter space is fully covered. Finally, all sub-regions are integrated to produce the complete set of scenario parameter combinations for full coverage.

In order to facilitate the computation of parameter space coverage and reduce the associated computational burden, the logical scenario parameter space is first discretized, resulting in a set of *n*-dimensional concrete scenario parameter combinations, as defined in Equation (6).

(6) $$G=\left\{\left.x\in {\mathbb{R}}^{n}\right|x⊘\Delta \in {\mathbb{Z}}^{n}\right\}$$

In this formulation, *x* denotes a discretized grid point within the parameter space, ∆∈ℝ_+_*^n^* denotes the discretization step in each dimension. When selected as a scenario test point, its associated coverage radius *r_i_*∈ℤ_+_*^n^* can be derived from the scenario representativeness model, where *r_i_* = [*r_i_*^(1)^, *r_i_*^(2)^, …, *r_i_*^(n)^] specifies the coverage distance along each dimension.

The greedy algorithm for generating scenario parameter combinations within the parameter space is guided by the concept of scenario heat. Scenario heat is defined as the distance between a given concrete scenario and the estimated boundary of human driving capability. Since the primary objective of conventional testing is to identify the performance limits of the system under test, the proposed greedy algorithm prioritizes parameter regions with high scenario heat. The computation of scenario heat *h* is shown in Equation (7). Equation (8) is the result computed from per-dimension normalized scenario parameters.

(7) $$h_{0}\left(x_{i}\right)=\exp\left(-\alpha \times \mathrm{dist}\left(x_{i}{,\Psi }_{0}\right)\right)$$

(8) $$\mathrm{dist}\left(x_{i}{,\Psi }_{0}\right)={\left({\left(x_{1}-x_{1}^{\prime }\right)}^{2}+{\left(x_{2}-x_{2}^{\prime }\right)}^{2}+\cdots +{\left(x_{n}-x_{n}^{\prime }\right)}^{2}\right)}^{\frac{1}{2}}$$

In this equation, *x_i_* denotes an uncovered parameter point within the scenario parameter space, and Ψ_0_ represents the predefined human driving capability boundary, determined based on expert knowledge or empirical data. The term *x_i_* refers to the nearest point on *Ψ*_0_ to *x_i_*. The parameter *α* is a positive scalar controlling the decay rate of scenario heat with respect to distance from the boundary. By adjusting the value of *α*, the sensitivity to boundary proximity can be strengthened or weakened. In this study, *α* = 1 is used.

The objective of the H-GCO algorithm is to ensure that every discretized grid point in the parameter space is covered by at least one scenario parameter combination. This condition is formally defined in Equation (9).

(9) $$N\left({x,r}_{i}\right)=\left\{\left.y\in G\right|\forall j\in \left[1,n\right],\left|x_{j}-y_{j}\right|\leq {r_{i}}^{\left(j\right)}\right\}$$

In this equation, *N*(*x*,*r_i_*) denotes the discrete neighborhood centered at point *x* with a coverage radius *r_i_*. For any grid point *y* in the parameter space, if the distance between *y* and *x* in every dimension does not exceed the corresponding radius threshold, *y* is considered to be covered by the scenario parameter combination *x*. The set of all such points *y* constitutes the neighborhood *N*.

**Figure 5:**
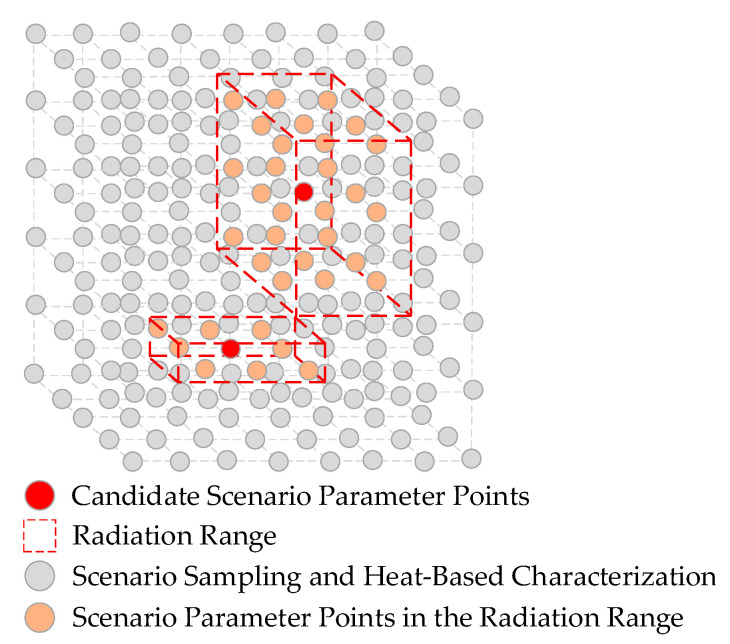
Geometric Illustration of Domain for Heat of Candidate Scenario Parameters.

During each iteration of the greedy selection process, based on the current heat value *h_k_*(*x_i_*), the sum of scenario heat in the neighborhood of each uncovered candidate parameter *x_i_* is calculated, as defined in Equation (10). A geometric illustration of this process is shown in Figure 5.

(10) $$\hat{s}\left(x_{i}\right)=\sum_{y\in N\left(x_{i}{,r}^{\mathrm{est}}\right)}h_{k}\left(y\right)\cdot \left(1-c\left(y\right)\right)$$

In this equation, *r^est^* defines the radius used for computing the neighborhood heat sum and is set to 5 in this study. The variable *k* denotes the current iteration of the greedy algorithm, and *c*(*y*)∈{0,1} is a binary coverage indicator function, where *c*(*y*) = 1 indicates that the point *y* in the parameter space has already been covered. At each iteration, the algorithm selects the parameter point with the maximum marginal heat gain as the next scenario to be added, as specified in Equation (11).

(11) $$x^{*}=\mathrm{argmax}\hat{s}\left(x_{i}\right)$$

Upon selecting the optimal parameter combination x*, a corresponding test scenario is instantiated, and its risk level is assessed using the surrogate model. Combined with the probability density at the selected point, the true scenario representativeness r* is derived. This value is then used to update the coverage indicator status for all parameter points within the actual coverage region of x*, such that c*(*y*) = 1.

As the greedy search iterates and the surrogate model conducts evaluations, the high-fidelity simulation test collision outcomes associated with the current parameter combinations are progressively revealed. In this process, the prior capability boundary derived from data increasingly fails to capture the uncertainty of collisions with sufficient accuracy. To address this limitation, it is necessary to refine the boundary function by incorporating a limited set of verified test points and their corresponding risk values. A structure-function-based refinement approach is proposed in this work. This method constructs a continuous structural function on top of the initial prior boundary and leverages newly acquired test points along with their actual risk parameters to perform localized consistency checks and apply real-time updates to the boundary estimate.

A symbolic distance function is first constructed based on the initial prior boundary that separates collision and non-collision regions, as shown in Equation (12).

(12) $$d\left(x\right)=\left\{\begin{array}{c} +\min_{z\in \Psi }\left\|x-z\right\|,\;x\in \mathrm{safety}\mathrm{zone}\\ -\min_{z\in \Psi }\left\|x-z\right\|,\;x\in \mathrm{collision}\mathrm{zone} \end{array}\right.$$

In this equation, *z* denotes a sampled point on the capability boundary.

Based on the distance function, a structural function is then established, as defined in Equation (13).

(13) $$W\left(x\right)={\left(1+\exp\left(-\delta \times d\left(x\right)\right)\right)}^{-1}$$

In this equation, *δ* denotes the smoothness coefficient, which regulates the degree of variation of the structural function in the vicinity of the boundary; in this study, *δ* = 1. When *d*(*x*) = 0, indicating that the parameter point lies exactly on the collision–safe boundary, the structural function takes the value *W*(*x*) = 0.5, representing the boundary condition. As the parameter point moves farther from the boundary, the value of *W*(*x*) smoothly approaches the representations of either high-risk or safe scenarios.

A risk–structure mapping function *R*(*ζ*) is constructed to map the observed real-world scenario risk index *ζ* to an expected value within the structural space.

(14) $$R\left(\zeta \right)=\exp\left[-\gamma \left(1-\zeta \right)\right]$$

In this equation, *γ* is a positive constant that governs the nonlinearity of the risk-level mapping.

During the *k* + 1 iteration of the greedy algorithm, the structural deviation at the parameter point *x_k_*_+1_ is computed as shown in Equation (15).

(15) $$\Delta_{k+1}{=W}_{k}\left(x_{k+1}\right)-R\left(\zeta_{k+1}\right)$$

In this equation, *W_k_*(*x_k_*_+1_) denotes the structural value assigned to the parameter point *x_k_*_+1_ by the current structural function, while *R*(*ζ_k_*_+1_) represents the expected structural value corresponding to the observed scenario risk level. A positive deviation indicates an overestimation, whereas a negative deviation reflects an underestimation of the structural value. To enable localized refinement of the structural function within the parameter space, a Gaussian kernel centered at *x_k_*_+1_ is introduced:

(16) $$K\left({x,x}_{k+1}\right)\;=\;\exp\left(-\varepsilon \times {\left\|x-x_{k+1}\right\|}^{2}\right)$$

In this equation, *ε* denotes a positive constant representing the kernel bandwidth, which controls the extent of the influence region; in this study, *ε* is set to 1. The term *K*(*x*, *x_k_*_+1_) indicates the influence intensity exerted by the test point *x_k_*_+1_ on the point *x* located on the capability boundary.

Accordingly, the structural function update can be expressed as:

(17) $$W_{k+1}\left(x\right){\;=\;W}_{k}\left(x\right)-\phi \times \Delta_{k+1}\times K\left({x,x}_{k+1}\right)$$

In this equation, *ϕ* denotes the step size parameter that governs the magnitude of adjustment in each iteration; in this study, *ϕ* is set to 0.5.

The boundary update is reflected in the shift in the structural function’s level set, whereby the boundary function is refined through the updated structural function, as expressed in Equation (18).

(18) $$\Psi_{k+1}=\left\{\left.x\in {\mathbb{R}}^{n}\right|W_{k+1}\left(x\right)=0.5\right\}$$

Accordingly, the scenario criticality of each uncovered parameter point can be recalculated, enabling the next iteration of greedy search to proceed. This process continues until all parameter points within the logical scenario space are fully covered.

### 4.2. Compressed Optimization of Scenario Parameter Combinations

The preliminary set of scenario parameter combinations achieving full coverage of the logical scenario parameter space is generated using the heat-guided hierarchical greedy algorithm. Although this method ensures complete coverage across all subregions, the inherently local nature of the greedy strategy often leads to redundancy in the selected test points. To further reduce testing costs and optimize the configuration of the point set, compression is required without compromising coverage integrity. Accordingly, a genetic algorithm is introduced to eliminate redundant test points and produce a more compact yet fully representative test scheme.

The genetic algorithm, inspired by natural selection and genetic inheritance, iteratively evolves improved solutions through selection, crossover, and mutation. The process is initialized with the complete scenario parameter set obtained from the hierarchical greedy algorithm:

(19) $$Q_{0}\;=\;\left\{q_{1}{,q}_{2},\cdots {,q}_{m}\right\}{\;q}_{i}\in {\mathbb{R}}^{n}$$

In this equation, *m* denotes the number of preliminary scenario parameter combinations.

The chromosome is defined as:

(20) $$g\;=\;\left(g_{1},g_{2},\cdots ,g_{m}\right)\;{\;g}_{i}\in \left\{0,1\right\}$$

In this equation, *g* represents a feasible configuration of the test point subset, where *g_i_* = 1 indicates that the corresponding test point *q_i_* is retained, *g_i_* = 0 indicates its removal. The initial population is generated by randomly excluding a portion of test points from *Q*_0_.

The optimization objective of the genetic algorithm is to minimize the number of retained test points, with the objective function defined as in Equation (21).

(21) $$\min f\left(g\right)=\sum_{i=1}^{m}g_{i}$$

In this equation, the fitness function *f(g)* corresponds to the “genetic length” of an individual, representing the number of retained test points. To ensure full coverage of the discretized parameter space, a feasibility constraint is introduced stating that every parameter point *x_j_* in the complete set *G* must be covered by at least one retained test point *q_i_*. The constraint is formally expressed as:

(22) $$\forall x_{j}\in G,\;\exists {i:g}_{i}\;=\;1,\;\left\|q_{i}-x_{j}\right\|\leq r_{i}$$

Any chromosome that fails to satisfy the aforementioned coverage constraint is directly eliminated from the population.

First, individuals are selected from the current population based on their fitness values using the roulette wheel selection method. Individuals with lower fitness values, which indicate fewer retained parameter combinations, are more likely to be selected as parents. Subsequently, single-point crossover is performed between the selected parent chromosomes according to a predefined crossover probability *p_g_*, set to 0.6. For two parent chromosomes *g*^(1)^ and *g*^(2)^, a crossover point *k*∈{1, 2, …, *m*−1} is randomly chosen, and gene segments are exchanged at this point to generate two offspring chromosomes:

(23) $$g^{\prime \left(1\right)}=\left({g_{1}}^{\left(1\right)},\cdots {{,g}_{k}}^{\left(1\right)}{{,g}_{k+1}}^{\left(2\right)},\cdots {{,g}_{m}}^{\left(2\right)}\right)$$

(24) $$g^{\prime \left(2\right)}=\left({g_{1}}^{\left(2\right)},\cdots {{,g}_{k}}^{\left(2\right)}{{,g}_{k+1}}^{\left(1\right)},\cdots {{,g}_{m}}^{\left(1\right)}\right)$$

Each offspring chromosome generated through crossover is subsequently subjected to mutation. With a mutation probability *p_m_* (set to 0.01 in this study), each gene *g_i_* undergoes independent mutation based on the following probabilistic rule:

(25) $${g_{i}}^{\prime }=\left\{\begin{array}{c} 1-g_{i}{,\;\mathrm{probability}:p}_{m}\\ {\;g}_{i}{,\;\mathrm{probability}:1-p}_{m}\; \end{array}\right.$$

Since crossover and mutation operations may compromise the completeness of parameter space coverage, each newly generated individual is subsequently subjected to a feasibility check, and a repair procedure is executed if the full-space coverage constraint is not satisfied. The repair strategy follows a greedy replenishment principle, in which the minimum number of original sampling points are selected from the uncovered grid to restore feasibility.

At each generation, the individual with the best fitness is retained to ensure a monotonically improving solution process. The algorithm terminates either when the maximum number of generations is reached or when no further improvement is observed over successive iterations. The compressed and optimized set of scenario parameter combinations corresponding to the best individual, denoted as Q*, is then returned as the final test set.

The process of method is as follows (Algorithm 1).
**Algorithm 1:** Process of method **Input:** logical scenario space G; discretization steps; naturalistic statisics(μ,∑); surrogates {LSTM (longitudinal), lane-change potential U, MCTS (lateral)}; parameters *ω*, *σ*, *λ*, *τ*, *ψ, r*_min_, *r*_max_, *r*_est_, *ϑ*, *η, α*, *δ*, *γ*, *ε*, *ϕ.***Procedure****1. Initialization**(1) Discretize each parameter dimension according to discretization steps to form the grid *G*.(2) Initialize the coverage indicator *c*(*x*) = 0; create an empty candidate set *Q*_0_.(3) Set the prior boundary and the associated structural function *W*(*x*).(4) Compute the initial heat map over *G* by *h*(*x*).**2. Heat-guided hierarchical greedy coverage optimization**Repeat until *c*(*x*) = 1 for all *x*∈*G*:(1) Compute the heat score *s*^(*x*) and select the maximum *s*^(x*).(2) Compute the probability index *A*(x*) and the scalar risk *ξ*(x*) from the surrogates.(3) Compute the per-dimension representativeness radius r(x*).(4) Set c*(*y*) = 1; append x* to *Q*.(5) Update the structural function and re-extract the boundary *W*(*x*) = 0.5.(6) Recompute *h*(*x*) for all uncovered *x*∈*G*.**3. Genetic algorithm under the full-coverage constraint**Iterate until convergence or a maximum number of generations:(1) Use the full coverage set *Q* obtained from the H-GCO stage as the initial solution.(2) Minimize the number of retained points, there must exist at least one retained *q_i_* satisfying the coverage condition for each *x*∈*G*.(3) Apply roulette-wheel selection, single-point crossover, bit-flip mutation and elite preservation.**Output:** Q*—a minimal set of concrete scenarios fully covering the discretized *G*.

## 5. Experiment

**Figure 6:**
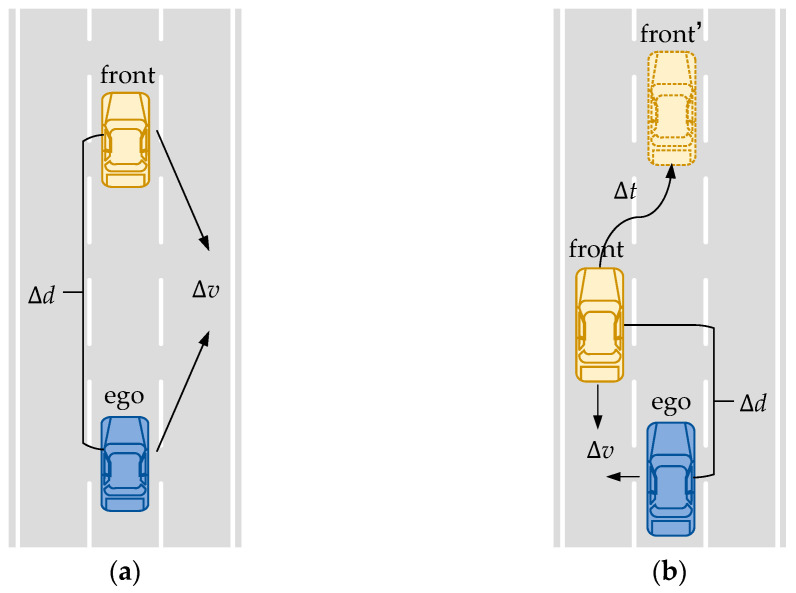
(**a**) Illustration of the Lead Vehicle Braking Scenario. (**b**) Illustration of the Lead Vehicle Cut-in Scenario.

The HighD public dataset is utilized in this study to extract two representative testing scenarios: the lead vehicle braking scenario and the lead vehicle cut-in scenario, as shown in Figure 6.

The parameter space for the braking scenario includes the initial relative velocity Δ*v* within the range [−10, 10] m/s and the initial relative distance Δ*d* within the range [5, 75] m. For the cut-in scenario, the parameter space consists of the initial relative velocity Δv within the range [−10, 20] m/s, the initial relative distance Δ*d* within the range [10, 100] m, and the cut-in stabilization time Δ*t* within the range [2, 5] s.

With the aim of generating concrete scenarios, the logical scenario parameter space is discretized into a grid. For the leading vehicle braking scenario, the relative distance and relative velocity between the ego vehicle and the leading vehicle are discretized using step sizes of 1 m and 1 m/s, respectively, resulting in a total of 1491 concrete scenario parameters to be covered. For the vehicle cut-in scenario, the initial relative velocity, initial relative distance, and cut-in stabilization time are discretized with step sizes of 2 m/s, 2 m, and 0.2 s, respectively, yielding a total of 11,776 concrete scenario parameters.

Based on statistical results derived from the HighD naturalistic driving dataset, the driver capability boundaries are predefined for the two types of scenario parameter spaces. The probability distribution of each scenario point within the parameter space is approximated using Equation (2).

### 5.1. Experiment of Test Scenario Coverage Ratio

**Figure 7:**
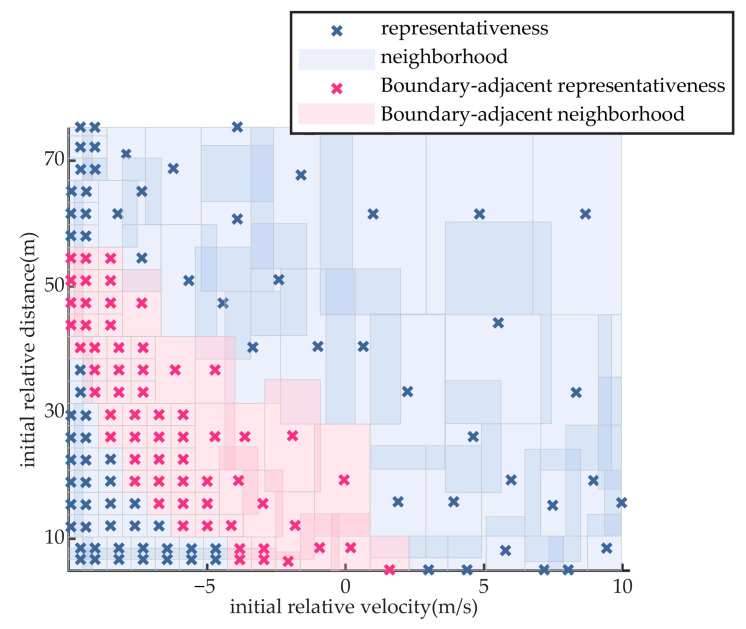
Extraction Results of Scenario Parameters for Braking Scenario.

**Figure 8:**
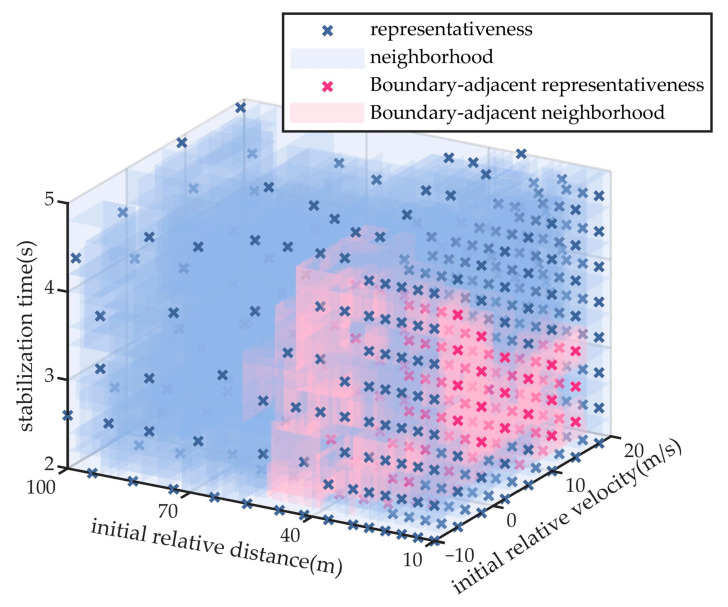
Extraction Results of Scenario Parameters for Cut-in Scenario.

The proposed scenario compression and optimization method is employed to select representative concrete scenarios from the parameter space. In the leading vehicle braking scenario, the weight coefficients for relative velocity and relative distance in the influence radius analysis are set to 0.6 and 0.4, respectively. For the vehicle cut-in scenario, the weight coefficients for relative velocity, relative distance, and cut-in stabilization time are assigned as 0.4, 0.3, and 0.3, respectively. The representative scenario parameters and their corresponding neighborhood coverage results for the two types of scenario generation are illustrated in Figure 7 and Figure 8. Among them, scenario parameter points near the performance boundary of the automated driving system are highlighted with distinct colors.

For the lead vehicle braking scenario, the proposed method extracts 141 representative test scenarios, resulting in a reduction of over 91% in testing cost compared to exhaustive full-space evaluation involving 1491 scenarios. For the lead vehicle cut-in scenario, 482 test scenarios are selected using the proposed approach, reducing the testing cost by more than 96% relative to the 11,776 scenarios required by full-space enumeration.

As illustrated in Figure 7 and Figure 8, the scenario neighborhood sets extracted by the proposed method successfully encompass all specific scenarios within the parameter space, thereby achieving complete coverage of the logical scenario parameter space.

To further validate the advantages of the proposed method in testing efficiency and comprehensiveness, comparative experiments were conducted within the same logical scenario parameter space against two scenario generation methods commonly used by third party testing organizations, namely monte carlo and combinatorial testing, as well as an accelerated search algorithm for hazardous scenarios based on importance sampling [28].

The monte carlo method, grounded in probabilistic statistics and random sampling, is widely used in automated driving validation for efficiently generating large-scale test scenarios and estimating their distributional characteristics. Combinatorial testing, based on interaction coverage principles, systematically selects variable combinations to capture parameter interactions with a minimal number of test samples, and is typically used to construct representative scenario subsets. Importance sampling method allocates limited test samples to target regions by constructing a guided sampling distribution and is commonly used for accelerated search of hazardous scenarios and estimation of critical event probabilities. This study likewise uses hazardousness and the probability to define the target density and constructs the proposal distribution accordingly, as shown in Equation (26).

(26) $$e_{t}\left(x_{i}\right)=E\left(x_{i}\right)\xi_{t}\left(x_{i}\right)/\sum_{i=1}^{n}E\left(x_{i}\right)\xi_{t}\left(x_{i}\right)$$

In this equation, *t* denotes the iteration index, *ξ_t_*(*x*) is a continuous risk measure derived from the risk labels *ξ* via ordinal logistic regression, as specified in Equation (27).

(27) $$\xi_{t}\left(x_{i}\right)=\sum_{j=1}^{5}{\mathrm{vector}}_{j}\pi_{t,j}\left(x_{i}\right)$$

In this equation, *vector* denotes the ordered vector of risk labels *ζ*, and π*_t_*_,*j*_(*x*) is obtained via maximum-likelihood training.

**Figure 9:**
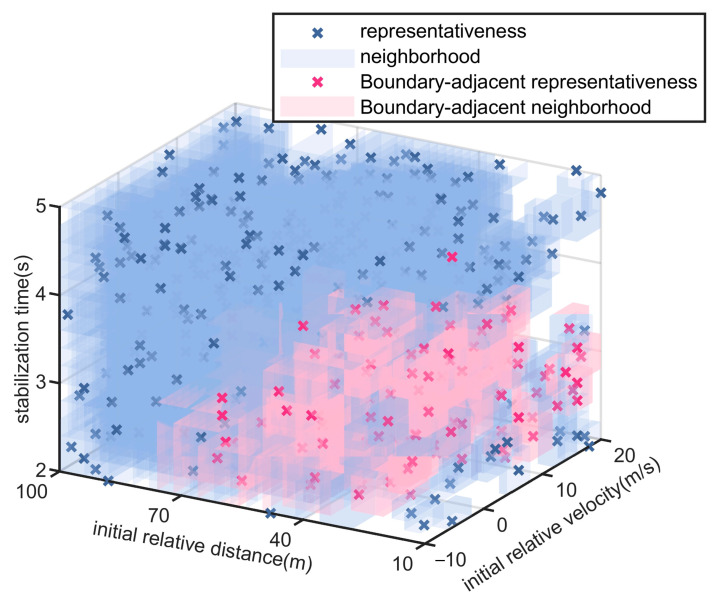
Extraction Results of Scenario Parameters for Monte Carlo Method.

**Figure 10:**
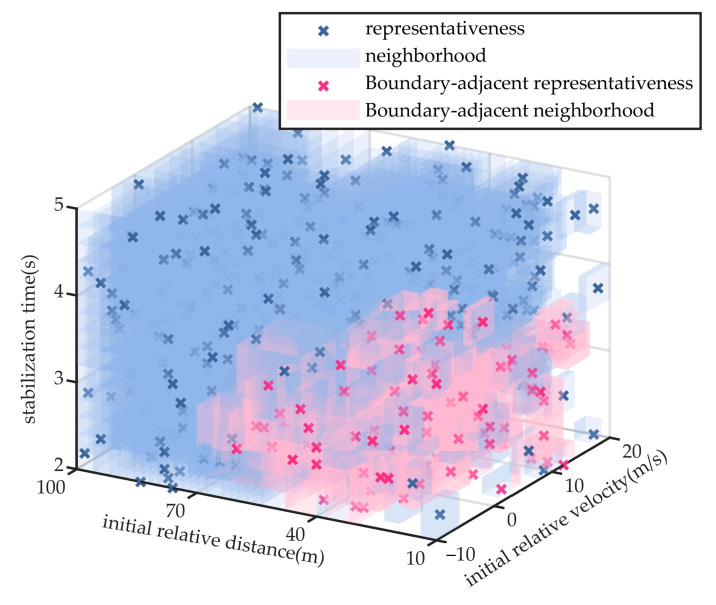
Extraction Results of Scenario Parameters for Combinatorial Testing Method.

**Figure 11:**
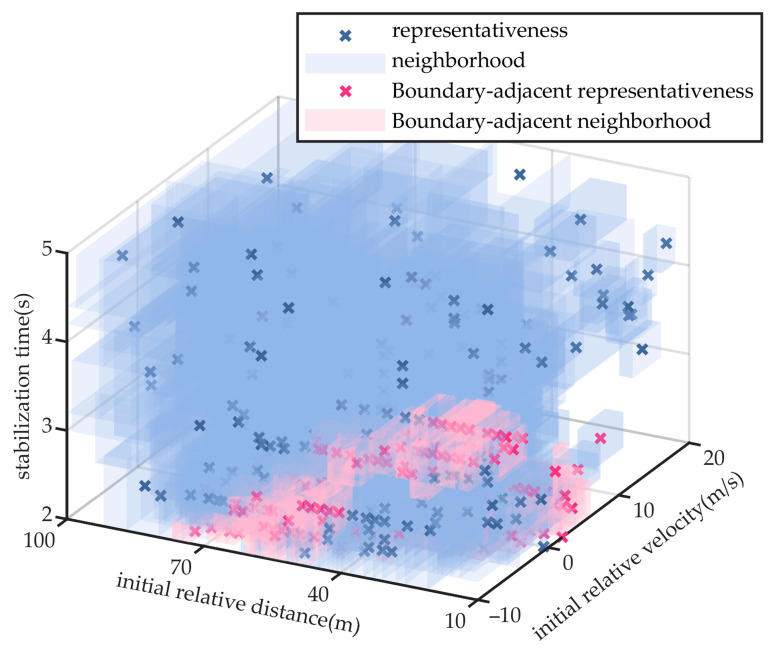
Extraction Results of Scenario Parameters for Importance Sampling Method.

The vehicle cut-in scenario is used for comparative analysis. To ensure reproducibility and consistency, both the monte carlo and combinatorial testing methods extract 482 test scenarios, and the sample size in the final round of the importance sampling method was constrained to ensure that the iteration terminated upon extracting 482 test scenarios, matching the number used in the proposed method. All four methods apply the same neighborhood-based scenario coverage representation to facilitate objective performance comparison. The extracted scenario parameters and their respective coverage distributions for the three baseline methods are shown in Figure 9, Figure 10 and Figure 11. The scenario coverage results obtained by the four scenario generation methods are presented in Table 3.

Under the condition that all methods generate an equal number of test scenarios (482) and aim to cover the same total number of specific scenarios (11,776), the neighborhood regions generated by the monte carlo method cover 9926 specific scenarios, yielding a coverage rate of 84.3%. The combinatorial testing method achieves coverage of 10,185 specific scenarios, corresponding to a rate of 86.5%. The importance sampling method achieves coverage of 8473 specific scenarios, corresponding to a rate of 72.0%. Neither method succeeds in achieving full coverage of the scenario parameter space.

### 5.2. Experiment of Performance Boundary Fitting Accuracy

Taking the cut-in scenario as a representative case, Figure 8, Figure 9, Figure 10 and Figure 11 indicate that the scenarios selected by the proposed method and importance sampling method are concentrated in high-risk regions of the parameter space (regions characterized by smaller initial relative distances), which conforms to the general principle of increasing test frequency for high-risk cases. In contrast, the scenarios generated by the monte carlo method and the combinatorial testing method exhibit a noticeably uniform distribution, which does not align well with the distribution of scenario importance. This discrepancy leads to a limitation in the ability of uniform sampling methods to accurately fit the performance boundary of the system under test. However, importance sampling method tends to over-concentrate on scenarios that are both high-risk and highly probable, under-sampling the remainder of the parameter space and thereby yielding markedly insufficient coverage.

**Figure 12:**
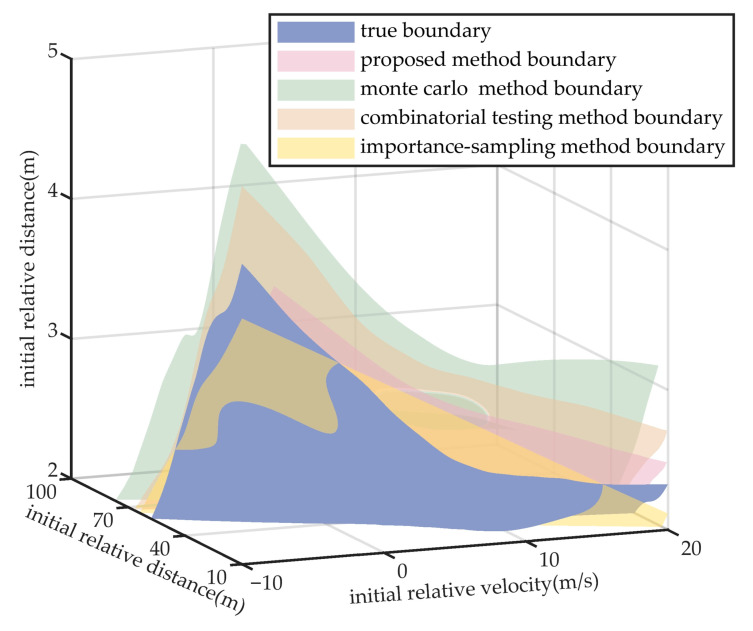
Illustration of Performance Boundary Fitting in Cut-in Scenario.

Furthermore, in order to quantify the difference in scenario effectiveness, a co-simulation platform based on Python 3.9, PreScan 2021.3, and CarSim 2020.0 is established to obtain the test results. The truth boundary of the system is derived by fitting 193 parameter points located near the collision–noncollision division (points with risk indices corresponding to minor collision and near-collision) identified in the previous exhaustive testing results. These scenarios and their parameter locations are used as sampling points to calculate the RMSE between the true system boundary and the boundaries generated by the four scenario generation methods, which serves as the quantitative evaluation metric. As the parameter dimensions differ in units and scales, they are normalized to the [0, 1] range to eliminate the influence of scale differences [29]. The boundaries are illustrated in Figure 12, with the corresponding RMSE values summarized in Table 4.

Results indicate that the proposed method yields a more accurate approximation of the performance boundary. This advantage stems from the concentration of test scenarios in high-risk areas, where a greater number of scenario parameter points are located near the actual boundary. As a result, the proposed method reduces the RMSE by 11% compared with the monte carlo method and by 6% compared with the combinatorial testing method. Similarly, owing to its accelerated-search property, the importance-sampling method demonstrates competitive boundary-fitting accuracy.

## 6. Conclusions

Aiming to address the challenges of low coverage and limited consistency in the sampling process of logical scenario parameter spaces, this study proposes a comprehensive scenario generation method for automated driving system testing, based on scenario representativeness. By introducing a quantitative evaluation of scenario representativeness and formulating the optimization problem as a differentiated full-coverage solution in the sample combination space, the proposed approach enables complete coverage of the logical scenario parameter space with a reduced number of concrete test scenarios.

Comparative experiments using cut-in and car-following braking scenarios demonstrate that the proposed method outperforms existing techniques in terms of scenario coverage, testing efficiency, and performance boundary fitting accuracy. Furthermore, by adjusting element weights, the method can be extended to support higher-dimensional scenario spaces. The generation of a unified and standardized scenario library ensures consistency and reproducibility in the testing process, offering significant engineering value for third-party certification bodies seeking to establish standardized scenario-based testing frameworks.

## Figures and Tables

**Table 1:** LSTM Model Configuration.

Parameter	Data
Input Feature Dimension	5
Output Feature Dimension	1
LSTM Hidden Units	64
Number of LSTM Layers	3
Learning Rate	0.001
Dropout	0.1
Number of Epochs	20
Batch Size	8
Attention Type	/

**Table 2:** Parameter Settings of the Lane-changing Potential Field Model.

Parameter Type	Parameter Value
*I*	0.001
*M_i_*	5000 kg
*l*_1_	1
*l*_2_	0.05

**Table 3:** Comparison of Scenario Coverage Rates Across Different Methods.

Scenario Generation Method	Number of Representativeness	Coverage Rates
proposed method	482	100%
monte carlo method	482	84.3%
combinatorial testing method	482	86.5%
importance sampling method	482	72.0%

**Table 4:** RMSE of Performance Boundary Fitting Across Methods.

Scenario Generation Method	RMSE
proposed method	0.08
monte carlo method	0.19
combinatorial testing method	0.14
importance sampling method	0.07

## Data Availability

Dataset available on request from the authors.

## Abbreviations

The following abbreviations are used in this manuscript:

ADS	Automated driving systems
TTC	Time to collision
LSTM	Long short-term memory
MCTS	Monte carlo tree search
RMSE	Root mean square error
H-GCO	Hierarchical greedy coverage optimization
